# Passive Double-Sensory Evoked Coherence Correlates with Long-Term Memory Capacity

**DOI:** 10.3389/fnhum.2017.00598

**Published:** 2017-12-14

**Authors:** Anna Horwitz, Erik L. Mortensen, Merete Osler, Birgitte Fagerlund, Martin Lauritzen, Krisztina Benedek

**Affiliations:** ^1^Department of Neuroscience and Pharmacology, University of Copenhagen, Copenhagen, Denmark; ^2^Center for Healthy Aging, University of Copenhagen, Copenhagen, Denmark; ^3^Department of Clinical Neurophysiology, Rigshospitalet - Glostrup, Glostrup, Denmark; ^4^Department of Public Health, University of Copenhagen, Copenhagen, Denmark; ^5^Research Center for Prevention and Health, Rigshospitalet - Glostrup, Glostrup, Denmark; ^6^Center for Neuropsychiatric Schizophrenia Research, Psychiatric Center Glostrup, Glostrup, Denmark; ^7^Center for Clinical Intervention and Neuropsychiatric Schizophrenia Research, Psychiatric Center Glostrup, Glostrup, Denmark

**Keywords:** EEG, gamma coherence, neurocognitive function, long-term memory, working memory, steady-state visual evoked potentials, aging

## Abstract

**HIGHLIGHTS**

- Memory correlates with the difference between single and double-sensory evoked steady-state coherence in the gamma range (Δ*C*).
- The correlation is most pronounced for the anterior brain region (Δ*C*_*A*_).
- The correlation is not driven by birth size, education, speed of processing, or intelligence.
- The sensitivity of Δ*C*_*A*_ for detecting low memory capacity is 90%.

Cerebral rhythmic activity and oscillations are important pathways of communication between cortical cell assemblies and may be key factors in memory. We asked whether memory performance is related to gamma coherence in a non-task sensory steady-state stimulation. We investigated 40 healthy males born in 1953 who were part of a Danish birth cohort study. Coherence was measured in the gamma range in response to a single-sensory visual stimulation (36 Hz) and a double-sensory combined audiovisual stimulation (auditive: 40 Hz; visual: 36 Hz). The individual difference in coherence (Δ*C*) between the bimodal and monomodal stimulation was calculated for each subject and used as the main explanatory variable. Δ*C* in total brain were significantly negatively correlated with long-term verbal recall. This correlation was pronounced for the anterior region. In addition, the correlation between Δ*C* and long-term memory was robust when controlling for working memory, as well as a wide range of potentially confounding factors, including intelligence, length of education, speed of processing, visual attention and executive function. Moreover, we found that the difference in anterior coherence (Δ*C*_*A*_) is a better predictor of memory than power in multivariate models. The sensitivity of Δ*C*_*A*_ for detecting low memory capacity is 92%. Finally, Δ*C*_*A*_ was also associated with other types of memory: verbal learning, visual recognition, and spatial memory, and these additional correlations were also robust enough to control for a range of potentially confounding factors. Thus, the Δ*C* is a predictor of memory performance may be useful in cognitive neuropsychological testing.

## Introduction

The aim of the present study was to investigate whether memory is associated with functional brain connectivity, specifically whether measures of memory function correlate with neurophysiological gamma band coherence, using a novel two-sense stimulation method involving passive non-task monomodal and combined bimodal stimulation. We contribute to the literature by employing a method that can account for certain unobserved confounding factors by focusing on within-individual differences in coherence between two stimulation procedures. Our study provides evidence that the difference in electrophysiological connectivity between single-sensory and double-sensory stimulation is associated with long-term memory, accounting for a wide range of unobserved and observed potentially confounding factors.

Different theories have tried to explain how memory works when seen as a neural process. One starting point is the observation that communication between different brain regions provides the basis for the integration of, for example, sensory information and sensory-motor coordination, which are critical for information processing, learning, and memory (Herrmann et al., 2004a). For this reason alone, one may hypothesize that differences in brain connectivity between individuals may explain differences in memory performance. Consistent with this notion, the so-called Hebbian theory hypothesize how neurons connect to become engrams and bind together and store memory traces (Morris, 1999).

While previous studies have typically investigated cognitive function in relation to electrophysiological activity measured during an *active* memory task, this present study investigates whether a *passive*, multi-sensoric stimulation response compared to the monomodal response-level for a subject, can explain memory capacity.

Cerebral rhythmic activity and oscillations constitute an important pathway of communication between cortical cell assemblies (Gray et al., 1989; Gray and McCormick, 1996; Varela et al., 2001) and are key factors in perception and memory (Steriade et al., 1996; Başar et al., 2000). Several studies have found links between memory performance and brain oscillations at various frequencies including cross-frequency couplings (e.g., Klimesch, 1999; Başar et al., 2001; Howard et al., 2003; Jensen and Lisman, 2005; Vertes, 2005; Jensen et al., 2007; Hanslmayr et al., 2008, 2011, 2014; Siegel et al., 2009; Engel and Fries, 2010; Herman et al., 2013; Lisman and Jensen, 2013; Staudigl and Hanslmayr, 2013; Ekstrom and Watrous, 2014; Hanslmayr and Staudigl, 2014; Roux and Uhlhaas, 2014). Meanwhile, other studies have casted doubt upon the existence of oscillatory signatures that are uniquely associated with memory performance (Hanslmayr and Staudigl, 2014; Hanslmayr et al., 2014).

Short-term memory processes may be reflected in anterior limbic system oscillations, whereas long-term memory processes are reflected in posterior thalamic system oscillations (Klimesch, 1996).

It has long been unclear if the association between brain oscillations and cognitive function represents a fundamental causal link or just an epiphenomenon (Herrmann et al., 2013). Recently, however, transcranial alternating current stimulation has been used to support the notion that brain oscillations have causal effects on cognitive performance, indicating that the association between brain oscillations and cognitive processes are not just an epiphenomenon (Herrmann et al., 2013, 2016).

The connection between neurons are commonly thought to be reflected by the synchronicity between brain areas, which can be measured by coherence. Some studies have shown that both short and long term memory, as well as associative learning, is associated with brain synchronization (Klimesch et al., 1996, 1997a,b,c; Klimesch, 1997, 1999; Miltner et al., 1999; Fell et al., 2001). Alzheimer's patients display impaired performance when exposed to multiple tasks simultaneously in a dual-task paradigm (Baddeley and Della Sala, 1996). Furthermore, there is evidence of a loss of gamma band synchronization in Alzheimer's disease (Stam et al., 2002). Here, we investigated the correlation between the difference in a subject's gamma coherence (between a monomodal and bimodal stimulation) and long-term and working memory. As processes that modulate the occipital gamma activity reside in the frontal cortex (Knight et al., 1999; Barceló et al., 2000; Herrmann et al., 2004a,b; Muthukumaraswamy et al., 2010) and because some studies tend to find that prefrontal activity is particularly related to memory performance (see Paller and Wagner, 2002; for a review; Hanslmayr et al., 2008, 2011; Benchenane et al., 2011) we focused on both the total coherence and the anterior coherence.

Neuronal oscillations at high frequencies (e.g., in the gamma range) has been found to be highly involved in the establishment of synchronicity (Varela et al., 2001). High-frequency oscillations were first described in animals in 1942 (Adrian, 1942) and subsequently in humans in 1960 (Chatrian et al., 1960). The study of neural oscillations experienced a renaissance when it was found to correlate with perceptual binding (Gray et al., 1989; Singer and Gray, 1995; Pulvermüller et al., 1997; Engel et al., 2001). Gamma band activity may also be involved in memory and object recognition (Tallon-Baudry et al., 1998; Keil et al., 1999; Başar et al., 2000; Pesaran et al., 2002; Kaiser et al., 2003), attention (Tiitinen et al., 1993; Gregoriou et al., 2009), arousal (Strüber et al., 2000), linguistic processing (Pantev, 1995; Pulvermüller et al., 1995; Eulitz et al., 1996), associative learning (Miltner et al., 1999), consciousness and REM sleep (Keller et al., 1990; Llinás and Ribary, 1993), and other behavioral and perceptual functions (Revonsuo et al., 1997). However, gamma band activity is not unique to any of these functions and may not be a specific indicator of any of these processes (Herrmann et al., 2004b).

Gamma activity is affected by a subject's attention, alertness, and arousal. Therefore, we focus on the *difference* in a subject's coherence between the two stimulations, rather than the level of coherence in either stimulation, allowing us to automatically account for all factors that affect the coherence measures in a constant fashion^1^. Furthermore, we control for a range of potentially confounding variables that could affect stress level, attention, and alertness, such as mental fatigue and sleep quality index scores, in the time leading up to testing.

Memory has been shown to be related to brain activity at multiple frequencies (see e.g., the introduction to Hanslmayr et al., 2014; Roux and Uhlhaas, 2014). Since age tends to be an important factor for memory and brain oscillations (Duffy et al., 1984; Macpherson et al., 2009, 2012; Muthukumaraswamy et al., 2010; Stranahan et al., 2011; Gaetz et al., 2012; Jessen et al., 2015), and since our cohort consisted of elderly men, gamma band activity appeared to us as the most promising candidate frequency band for investigation. Furthermore, since this study is the first to investigate the association between the individual mono-to-bimodal difference in coherence and memory, we wanted to maximize the likelihood of detecting a possible correlation, rather than exploring a broader set of frequency bands. We therefore focused on a single frequency band (gamma), which allowed us to perform our measurements on a larger number of participants.

We hypothesize that a correlation exists between long-term memory capacity and the change in brain coherence in response to additional senses being stimulated simultaneously in a combined passive stimulation setup. This hypothesis is based on the idea that more brain connectivity is generated in response to additional stimuli, potentially due to decreased cortical inhibition, in subjects with lower memory capacity (Osipova et al., 2006; Hansen et al., 2014). Furthermore, this hypothesis is consistent with a notion that multi-sensory stimulation reveals more information about a subject's general level of cognitive function than a single-sensory stimulation. While not essential for our working hypotheses, we focus on gamma-band activity due to its association with both higher cognitive function and synchronicity.

## Materials and methods

### Participants

The present sub-sample was selected among participants from the Metropolit Cohort of males born in Copenhagen in 1953 (Osler et al., 2006). The participants of the Metropolit Cohort have been physically and mentally investigated throughout their lives. They therefore enable us to control for important potential confounding factors. The study is based on examination of 40 men (aged 61–62 years at the time of data collection). Initially, 45 subjects were investigated, but five were excluded from the analysis because of the use of a hearing aid. Furthermore, an additional four test persons were excluded from some of the additional robustness analyses due to missing data on birth measures. Finally, an additional observation is missing from one additional robustness analysis due to missing data on visual attention. Data for a subset of the subjects used in this analysis were used in Horwitz et al. (2017). All subjects had normal (or corrected-to-normal) vision and normal hearing based on detected hearing thresholds. Thirty-six out of forty participants were right-handed. The participants reported no previous history of neurological or psychiatric disorders and were neurocognitively examined using the cognitive tests described below.

The men had an average IST-2000-R score of 34.38 (*SD*: 11.24). They had on average underwent 13.23 (*SD*: 2.29) years of education. Their birth weight was on average 3,544 g (*SD*: 534 g) and their birth length was on average 52.58 cm (*SD*: 2.41 cm).

The magnetic resonance imaging scans were investigated for abnormalities by in-house radiologists. No participants suffered from cerebral atrophy. The subjects received no compensation besides lunch and transport to and from the hospital.

The Metropolit cohort was used for two main reasons. First, the cohort has been examined throughout life, making it possible to understand our findings in the context of other published information about the cohort and to control for variables measured earlier in life. Second, the cohort is homogenous with respect to age and location of birth, meaning that some background variables are held fixed. Third, we believe that focusing on elderly subjects is preferred due to the possible clinical relevance of detection of memory performance in age-related conditions such as cognitive decline and dementia.

There are two main reasons for focusing on elderly men. First, the clinical implications of EEG-memory correlations would be mainly relevant for elderly individuals. Second, focusing on a homogenous group of individuals, and therefore a single gender, may help maximizing statistical precision.

### Ethics statement

The study was approved by the local ethical committee (*De Videnskabsetiske Komiteer for Region Hovedstaden*) and registered by the Danish Data Protection Agency. All participants provided written informed consent.

### Procedure

For each subject, the entirety of the measurements were generally speaking conducted within a day. Subjects met at Rigshospitalet–Glostrup at 8 a.m. and left around 4 p.m. The measurements occurred in the following order. First, a blood sample was drawn from the subject. Second, the subject underwent cognitive testing with the following tests and in this order: Mini-mental state examination, Addenbrooke's cognitive examination, Trail Making A& B, Symbol-digit modalities test, 15 Word Pair, Cambridge Neuropsychological Test Automated Battery (Motor Screening Task, Spatial Recognition Memory, Pattern Recognition Memory, Stockings of Cambridge, Paired Associates Learning, Reaction Time, Rapid Visual Processing), 15 word pair recall, and finally the Intelligenz-Struktur-Test 2000-R. Third, the subject answered questionnaires on fatigue, sleepiness and depression. Fourth, the subjects were given lunch and had a break. Fifth, the subjects were measured with EEG and functional magnetic resonance imaging (the order of this changed with every other subject). The only deviations from completing all measurements within a day happened whenever the functional magnetic resonance imaging could not be performed on the day for practical reasons. When this happened, the subject was invited a second time to complete this measurement.

### Stimulation

We used two different stimulation designs. The first stimulation consisted of a single-sensory design that stimulated the visual system with a flicker rate of 36 Hz. The second stimulation consisted of a double-sensory design in which participants were stimulated simultaneously with an auditory (presented with a modulation frequency of 40 Hz) and visual paradigm (36 Hz) in a combined stimulation setup. In all stimulations, the order of presentation to the subjects was randomized.

The two-stimulation design is central to our empirical strategy. In particular, it enables us to investigate the difference in gamma coherence between the two stimulations. Thereby, we automatically account for a range of factors that may affect both measurements proportionally. For example, saccade-tendency, retina characteristics, electrode impedance, skull morphology, and other systematic measurement errors, should affect both measurements proportionally, and will therefore be differenced away. This empirical strategy will be further elaborated upon below.

We use a steady-state stimulation design. Steady state visual stimulation response can be provoked by flickering an image at a frequency above 3 Hz (Regan, 1989), and varies as a function of the temporal frequency of the driving stimulus (Rubin, 1915; Herrmann, 2001). Importantly, this method demonstrate a robust, higher signal-to-noise, and lower artifact ratios compared to the transient VEPs that are evoked at lower stimulation frequencies.

In general, the strongest steady-state response is obtained by stimulation frequencies at 10, 20, or 40 Hz (Herrmann, 2001). For the visual stimulation, we chose the technically largest possible flicker rate in the gamma range allowed by the refreshing rate of the computer monitor (36 Hz). Furthermore, our study design enable us to distinguish between the visual and the auditory brain responses by stimulating the senses at slightly different frequencies. Stimulating both senses at the same frequency would prevent distinguishing the response to either sensory stimulation.

#### Monomodal visual stimulation

For visual stimulation the subjects' were shown a flickering image (a black/white “Rubin's vase; Rubin, 1915).

**Figure 1:**
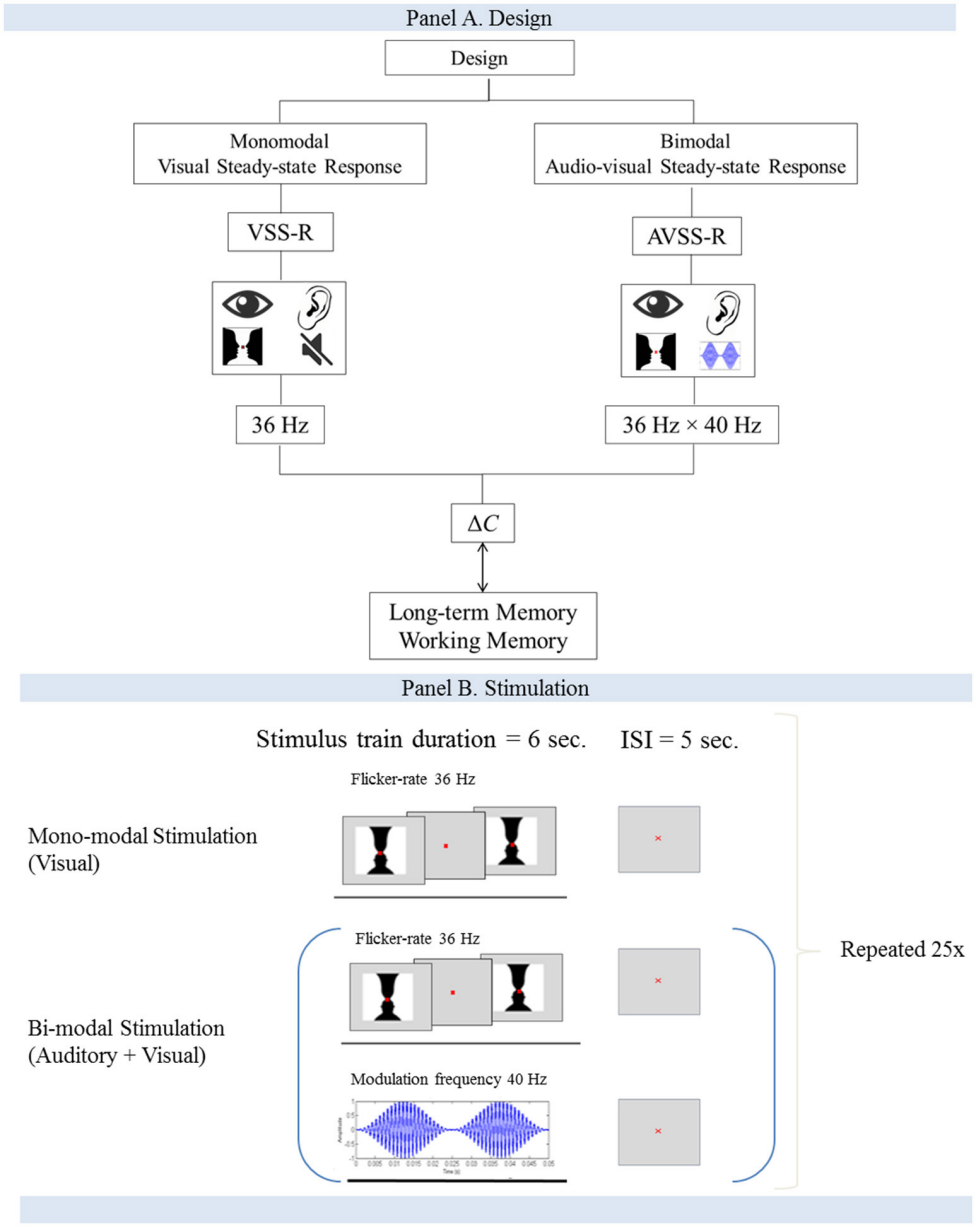
**(A,B)** Flow-charts for the study design and the stimulation procedure.

The size of the image was designed to span the central visual area and measured 5 degrees horizontal and 3.25° vertical (8.72 × 5.72 cm). Each stimulus consisted of an “on/off design” in which the image was interchanged with a gray background at a flicker rate of 36 Hz. The duration of a block was 6 s, with a 5-s inter-stimulus interval. A red fixation cross (0.33 mm) was present during the stimulation. This block of stimulation and inter-stimulation intervals was repeated 25 times (Figure 1).

#### Bimodal audiovisual stimulation

The double-sensory stimulation was performed by presenting the participants with both monomodal stimulations simultaneously (Figure 1B). The participants were exposed to an amplitude-modulated (AM) auditory stimuli (carrier wave 1 kHz, modulation frequency 40 Hz) and the “on/off” Rubin's vase image at a flicker rate of 36 Hz. The duration of a block was 6 s of stimulation, followed by a 5-s inter-stimulation interval. Each block was repeated 25 times.

### Data acquisition

A soft elastic cap with 64 surface electrodes (Ag/AgCl) was used (Quick-Cap, Compumedics), with the electrodes placed according to the international 10–20 system and connected to a bioamplifier (SynAmps, Compumedics). Neuroscan (Curry7.4 NeuroScan, Compumedics) was used to record signals and for data processing. The ground electrode was incorporated into the cap by the manufacturer over the midline frontal region and the reference placed between the Cz and Pz electrodes. In the data analysis the signals were re-referenced to bimastoid electrodes (M1, M2). We chose to reference the signal to the mastoid electrodes out of concern of clinical relevance. Mastoid referencing can yield reasonable results even in the case of few electrodes.

Electrode impedance was kept below 5 kΩ. Horizontal and vertical electro-oculographic data were registered with two bipolar channels. Two Electrocardiogram (ECG) electrodes were placed in the heart axis and two electrodes in a submental position to record EMG artifacts. EEG measurements were sampled at 2 kHz with an analog, anti-aliasing RC low-pass filter at 800 Hz. The EEG data were digitally high-pass and low-pass filtered offline with a Hann function filter at 0.5 Hz and 250 Hz and a tapering window at 10%. No notch filters were applied.

### Data preparation and analysis

The data were prepared as follows. Average event-related potentials (ERPs) were extracted from epochs of −500 to 6,000 ms relative to the stimulus onset, then baseline-corrected using the 500 ms interval before stimulus onset. Each epoch was extracted at one of the trigger events where a single image stimulus provoked phase-locked averaging (Figure 1B). As we were interested in visual stimulation and the difference between monomodal and bimodal stimulation, we investigated a narrow gamma band range from 33 to 38 Hz.

Coherence was calculated in CURRY 7.4 Neuroscan in three different time windows: 100, 1,000, and 6,000 ms. The 100 ms time window was used in the main analysis and the others in **Table 6**. The parameters that needed to be specified were the minimal distance between electrodes (50 mm), minimum lag (1 ms), and the maximum lag (2.5 ms). In all cases, we chose the standard value in CURRY 7.4 Neuroscan. Before analyzing the data, epochs were visually inspected for muscle artifacts, eye movements, ECG artifacts, and other detectable artifacts, which were then rejected using covariance methods. Data were also rejected if eye movement artifacts or electrode drifts were visible in the data plots.

### Artifacts

Initially, the data were visually inspected for obvious anomalies (such as an absence of signal), but none were found in any of the measurements. The data were then corrected offline for artifacts, eye movements, and ECG artifacts using the covariance method incorporated into the software CURRY 7.4 Neuroscan. Artifact-affected intervals associated with eye-related movements were identified using thresholds established with the vertical eye electrodes, with the lower and upper thresholds set at −200 and 200 μV, respectively. Voltages outside of this interval were considered indicative of eye-related movements, and measurements in all channels between −200 and 500 ms were considered to potentially be affected by artifacts. In a similar manner, we defined ECG -related artifact-affected intervals between −200 and 500 ms, surrounding QRS complex detection. The covariance method used in CURRY 7.4 Neuroscan to correct data in artifact-affected intervals involves covariance analyses between the artifact channel and each EEG channel, wherein linear transmission coefficients are computed for the basis of subtracting a proportion of the voltage from each data point in the artifact interval.

Furthermore, it should be noted that by using a differenced measure on the individual level, systematic measurement errors are automatically accounted for.

### Cognitive assessment

Global neurocognitive function was assessed initially with the Mini-Mental State Examination (MMSE) and Addenbrookes Cognitive Examination (ACE) to exclude possible signs of dementia. Indeed, as reported in the summary statistics in Table 1, the subjects scored within normal ranges. In addition, we used a number of paper tests of verbal memory and speed of processing, as well as five subsets with a focus on visual memory, executive function, and attention from the CANTAB battery.

**Table 1:** Characteristics of the sample and neurocognitive measures (*N* = 40).

	**Variable name**	**Mean**	***SD***	**Min**	**Max**
**ELECTROPHYSIOLOGICAL VARIABLES**					
Coherence measures	Δ*C_*A*_*	81.98	95.07	−109	249
	Δ*C_*P*_*	86.95	104.11	−136	311
	Δ*C_*AP*_*	127.15	214.73	−374	700
	Δ*C_*SI*_*	103.55	177.19	−290	569
**NEUROPSYCHOLOGICAL VARIABLES**					
Intelligence test	IST2000-R	34.38	11.25	8	54
Long-term recall	Verbal memory, recall	9.80	2.88	3	14
Working memory	Verbal memory, learning	30.48	9.49	9	42
	PAL (total errors multiplied by −1, adjusted)	27.34	26.06	4	86
Visual working memory	PRM (number correct)	21.15	1.96	16	24
	SRM (number correct)	16.70	1.62	12	20
Executive function, planning	SOC	8.73	1.93	4	12
Speed of processing	Trail-making Test A	33.60	10.30	18	61
	SDMT	45.43	9.02	28	72
Executive function	Trail-making Test B	77.55	23.52	45	148
Visual attention	RVP	0.93	0.04	0.83	0.99
**SOCIO-DEMOGRAPHIC VARIABLES**					
Years of education	Years of education	13.23	2.29	8	17
Birth weight (g)^#^	Birth weight	3, 544.44	533.69	2,200	4,600
Birth length (cm)^#^	Birth length	52.58	2.41	46.00	58.00

# *Number of Individuals*.

*PAL, Paired Associates Learning; PRM, Pattern Recognition Memory; SRM, Spatial Recognition Memory; SOC, Stockings of Cambridge*.

To assess long term-memory, we used a verbal memory test with recall of 15 word pairs after a 1-h interval between learning procedures (15 word-paired associates recall scores test). To assess working memory (Baddeley and Della Sala, 1996), we used five tests from the Cambridge Neuropsychological Test Automated Battery (CANTAB) with a focus on visual memory, executive function, and attention. CANTAB is a computerized neuropsychological assessment battery developed at Cambridge University in 1986 by Barbara Sahakian and Trevor Robbins (Sahakian et al., 1988; Fray et al., 1996; Robbins et al., 1998; Luciana and Nelson, 2002). CANTAB incorporates a wide variety of executive function, attention, and memory tasks to assess patterns of cognitive deficits and decline. CANTAB was originally intended for use as a general clinical neuropsychological test battery, and it has been used in a wide variety of clinical populations (Fray et al., 1996; Luciana and Nelson, 2002; De Luca et al., 2003; Levaux et al., 2007).

Intelligence was assessed using the Intelligence Structure Test 2000-R and speed of processing using the Trail-making A Test and Symbol-Digit Modalities Test (SDMT). The Trail-making Test A measures participants' subject's visual attention and provides information about visual search speed, scanning, and speed of processing while part B of the test measures mental flexibility, ability to switch tasks and is often used to assess executive functions, i.e., the capability to manipulate and update information, dual-task coordination, inhibition, and shifting processes. The SDMT measures patient attention, concentration, and speed of information processing (Giovagnoli et al., 1996; Sheridan et al., 2006). Speed of processing as measured with this test is also sensitive to detecting cognitive impairments.

For ease of comparison, we multiplied the Trail-making Test scores by −1, meaning that a higher score on this converted (“negated”) scale reflects higher cognitive function. Furthermore, we used attention as a control variable with the rapid visual processing (RVP) score, which is part of the CANTAB battery. The RVP test measures the ability to sustain visual attention.

The memory test battery included one verbal memory test, three visual memory tests, and one executive function test:

**Table:** 

***OUTCOME VARIABLES***	
*Long-term verbal recall*	15-word paired associates recall using number of correct answers on measured 1 h after the learning procedure
*Verbal learning memory*	15-word paired associates learning using the number of correct answers
*Visual memory, CANTAB*	Paired associates learning (PAL) assesses episodic memory and learning rate Pattern recognition memory (PRM) tests visual recognition memory for patterns Spatial recognition memory (SRM) tests recognition memory for spatial location
*Executive function, CANTAB*	Stockings of Cambridge (SOC)
***CONTROL VARIABLES***	
*Bio-social*	Birth length and weight Years of education
*Speed of processing*	Trail-making Test A SDMT
*Executive function*	Trail-making Test B
*Sleep habits and fatigue measures*	Multidimensional Fatigue Inventory (MFI-20) questionnaire Epworth Sleepiness Scale Pittsburgh Sleep Quality Index (PSQI)

We measured long-term verbal recall with a verbal test score: the number of correct answers on the 15-word paired associates recall measured 1 h after the learning procedure. We will at times refer to this at long-term memory capacity.

Furthermore, in a robustness analysis, we controlled for sleep habits as well as fatigue measures using the Multidimensional Fatigue Inventory (MFI-20) questionnaire (Smets et al., 1995). In controlling for sleep habits and sleep quality, we used the Epworth Sleepiness Scale and Pittsburgh Sleep Quality Index (PSQI).

### Definitions

The coherence between two channels (electrodes) x and y is defined as:

$$\begin{array}{l} {\text{c}}_{\text{xy}}=\frac{|{\text{G}}_{\text{xy}}{|}^{2}}{{\text{G}}_{\text{xx}}{\text{G}}_{\text{yy}}} \end{array}$$

where G_xy_ is the cross-spectral density between, respectively, channel x and channel y, and G_xx_ and G_yy_ are the autospectral densities of channel x and of channel y.

The coherence measure of a brain region is the number of coherent channel-pairs within that region, where a pair of channels are considered coherent if their coherence is above 0.8, i.e., if C_xy_ ≥ 0.8. In other words, the coherence measure of a set of electrodes in a brain region, *R*, is given by

$$\begin{array}{l} {\text{C}}_{\text{R}}=\underset{\text{x},\text{y}\in \text{R}}{\sum }{\text{I}\left({\text{c}}_{\text{xy}}\left(\text{f}\right)\right)}_{\geq 0.8} \end{array}$$

where x ≠ y and where I(*z*)_ ≥ 0.8_ is the indicator function, which is 1 if *z* ≥ 0.8 and 0 otherwise. We use the default threshold value of 0.8 used by Curry 7.4 Neuroscan. This threshold value reflects an attempt at balancing the trade-off between excluding channel-pairs that are not highly coherent, on the one hand, and allowing some degree of noise or otherwise unexplained divergence between the signals, on the other hand.

Note that since the number of possible electrode pairs within each region is a constant [equal to the binomial coefficient $\left(\frac{2}{k}\right)$ where *k* is the number of electrodes in the region], a change in the outcome measure therefore effectively measures a change in the ratio of coherent electrode-pairs to the number of possibly coherent pairs in the region.

The main variables of interests are defined as follows. The difference in total coherence was defined as:

$$\begin{array}{l} \Delta C_{T}=C_{\text{total},\text{ bimodal}}-C_{\text{total},\text{ monomodal}} \end{array}$$

where we have introduced a second subscript to *C*, indicating if the measurements originate from a monomodal or bimodal stimulation. Similarly, the difference in anterior coherence was defined as:

$$\begin{array}{l} \Delta C_{A}=C_{\text{anterior},\text{ bimodal}}-C_{\text{anterior},\text{ monomodal}} \end{array}$$

For ease of interpretation, we also worked with standardized versions of the variables given by μ_Δ*c*_*/*σ_Δ*c*_ where μ_Δ*c*_ is the average difference in coherence and σ_Δ*c*_ is the standard deviation (SD) of the difference in coherence.

### Statistical analysis

The main statistical analyses were performed using multiple linear regression models to investigate the relationship between cognitive function and gamma coherence. The linear regression model enabled us to estimate correlation between our outcome variable (i.e., the memory test score) and one or more explanatory variables (i.e., coherence and other variables), controlling for a range of factors.

We operate with a significance level of 5%. Meanwhile, coefficients with *p*-values between 5 and 10% were denoted as “significant at the 10% level.” Highlighting coefficients that are significant at the 10% significance level only is done to provide a more nuanced view on the statistical inference (see also the statement on *p*-values by the American Statistical Association, Wasserstein and Lazar, 2016). Confidence limits for proportions were calculated using Wald's method. Significance in the linear regression models was based on Eicker-Huber-White robust standard errors. As *t*-statistics and *p*-values are derived from the coefficient estimates and standard errors, we avoided redundancy by reporting estimated coefficients and standard errors in tables. The adjusted *R*^2^-values and semi-partial *R*^2^-values for the main explanatory variables were also reported in tabular form. All statistical analyses were performed in SAS 9.4.

## Results

### Long-term verbal recall and gamma coherence

All EEG measurements in this section relate to the anterior coherence and total coherence at 36 Hz i.e., the visual evoked response. As explained above, we focused on the difference between the monomodal and bimodal coherence (Δ*C*, i.e., *C*_*bimodal*_ − *C*_*monomodal*_). We also focused on the total coherence for the total brain as one unit, subdividing it into four units: anterior coherence (Δ*C*_*A*_), posterior coherence (Δ*C*_*P*_), anterior-posterior coherence (Δ*C*_*AP*_), and super-inferior coherence (Δ*C*_*SI*_). As shown in the summary statistics in Table 1, coherence increased on average in all regions, meaning that there were on average more coherent electrode-pairs in all regions during bimodal stimulation. In Table 2 we show the results of a “horse race” regression in which the four subunits were included as explanatory variables with both word-pair recall (columns 1–2), word-pair learning score (columns 2–4), and the four CANTAB measures, testing memory and executive function (columns 6–12) as outcome variables. Overall, long-term verbal recall correlated strongest with anterior coherence, as it had the largest absolute coefficient size and the highest explanatory power with a semi-partial *R*^2^ (Δ*C*_*A*_: semi-partial *R*^2^ = 0.29, *p* < 0.01; Δ*C*_*P*_: semi-partial *R*^2^ = 0.08, *p* = 0.08; Δ*C*_*AP*_: semi-partial *R*^2^ = 0.12, *p* = 0.01; Δ*C*_*SI*_: semi-partial *R*^2^ = 0.14, *p* < 0.01). Therefore, in the next part of the analysis we focused on the robustness of anterior coherence and total coherence with various specifications.

**Table 2:** Linear regression of the memory capacity and coherence for the four main regions of interest.

	**Long-term verbal recall**		**Learning score**		**PAL (total errors multiplied by −1, adjusted)**		**PRM (number correct)**		**SRM (number correct)**		**SOC (problems solved in min. move)**	
	**1**	**2**	**3**	**4**	**5**	**6**	**7**	**8**	**9**	**10**	**11**	**12**
Δ*C_*A*_*	−1.86^***^	−1.30^***^	−6.16^***^	−4.464^***^	−5.25	−1.57	−0.59^**^	−0.39	0.00	0.14	−0.67	−0.80^*^
	(0.45)	(0.31)	(1.83)	(1.16)	(4.72)	(4.15)	(0.24)	(0.27)	(0.33)	(0.26)	(0.40)	(0.40)
Δ*C_*P*_*	1.32^*^	1.63^***^	5.03^*^	5.85^**^	−3.82	−2.04	−0.43	−0.35	−0.17	−0.0	0.62	0.59
	(0.71)	(0.58)	(2.55)	(2.21)	(6.28)	(6.48)	(0.42)	(0.47)	(0.54)	(0.50)	(0.43)	(0.41)
Δ*C_*AP*_*	2.42^***^	2.98^***^	8.03^**^	9.72^***^	0.68	4.98	−0.71	−0.51	−0.95	−0.82^**^	0.10	0.32
	(0.83)	(0.67)	(3.52)	(2.78)	(11.47)	(9.02)	(0.61)	(0.60)	(0.80)	(0.73)	(0.68)	(0.65)
Δ*C_*SI*_*	−3.36^***^	−4.35^***^	−10.55^**^	−13.50^***^	0.13	−7.18	0.97	0.62	0.53	0.29	−0.44	−0.60
	(0.99)	(0.92)	(4.49)	(4.06)	(14.22)	(12.19)	(0.84)	(0.8)	(1.00)	(0.98)	(0.66)	(0.70)
Control variables	No	Yes	No	Yes	No	Yes	No	Yes	No	Yes	No	Yes
Semi-partial *R*^2^ Δ*C_*A*_*	0.29	0.23	0.27	0.22	0.03	0.00	0.06	0.03	0.00	0.01	0.10	0.13
Semi-partial *R*^2^ Δ*C_*P*_*	0.08	0.17	0.09	0.18	0.01	0.00	0.01	0.01	0.00	0.00	0.04	0.04
Semi-partial *R*^2^ Δ*C_*AP*_*	0.12	0.25	0.11	0.22	0.00	0.01	0.02	0.00	0.05	0.04	0.00	0.00
Semi-partial *R*^2^ Δ*C_*SI*_*	0.14	0.31	0.12	0.25	0.00	0.01	0.02	0.01	0.01	0.00	0.01	0.01
Number of individuals	40	40	40	40	40	40	40	40	40	40	39	39

*Robust standard errors in parentheses*.

* *p < 0.10*,

** *p < 0.05*,

*** *p < 0.01. All specifications include a constant that was omitted from the table*.

*Linear regression of the memory capacity and coherence for the four main regions of interest. “Control Variables” refer to the control variables of column 6 in Table 1. Columns 1–2 show a “horse race” regression with long-term memory and all four regions of interest included in the same model; the difference in anterior coherence remained significantly negatively correlated with long-term memory capacity and with a higher explanatory power compared to the other brain regions. Columns 3–12 show the “horse race” regression with the four regions of interest and different working memory test. PAL, Paired Associates Learning; PRM, Pattern Recognition Memory; SRM, Spatial Recognition Memory; SOC, Stockings of Cambridge*.

#### Difference in monomodal and bimodal coherence and long-term verbal recall

**Figure 2:**
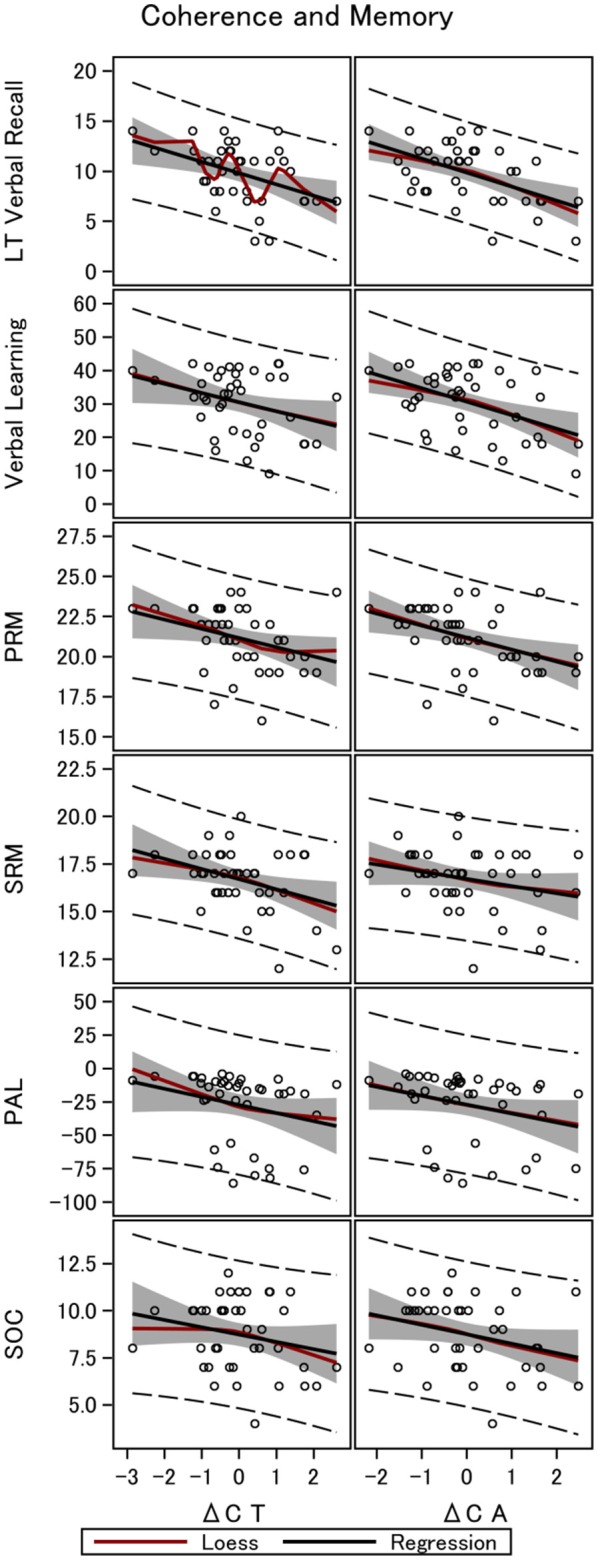
Gamma coherence and memory in 40 men. The figure shows the linear fit between long-term memory and four working memory tests, and the difference in coherence between monomodal and bimodal stimulation. Data are shown for the total brain coherence (Δ*C*_*T*_) and coherence in the anterior part of the brain only (Δ*C*_*A*_). LT Verbal Recall, Long-Term Verbal Recall Memory; Verbal Learning, Verbal Learning Memory; PRM, Pattern Recognition Memory; SRM, Spatial Recognition Memory; PAL, Paired Associates Learning; SOC, Stockings of Cambridge.

We found a negative correlation between the difference in total coherence for all electrodes which was most pronounced for the anterior part of the brain, between bimodal and monomodal visual stimulation (Δ*C*_*A*_*)* and long-term verbal recall. The raw correlation is depicted in Figure 2. Statistical analyses that account for a range of variables and show the same robust relationship are presented in Table 3. In particular column 1 of Table 3A, establish that Δ*C*_*A*_ was significantly negatively correlated with long-term verbal recall measured with the 15-word paired associates recall scores 1 h after learning and 15-word paired associates learning, all measured by the number of correct answers. The parameter estimate was −1.40, meaning that an increase in Δ*C*_*A*_of 1 *SD* was associated with 1.40 fewer correct answers in the memory recall test (semi-partial *R*^2^ = 0.28, *p* < 0.0001; Figure 2). The correlation is robust enough to control for speed of processing as examined with Trail-making Test A (column 2), frontal executive function as measured with the and Trail-making B (column 2). When controlling for subject's intelligence score (column 4) we found that the coefficient on the variable of interest (Δ*C*_*A*_) dropped in absolute size but remained negative. Furthermore, when including the word-pair learning score (column 5) in addition to memory recall, the difference in coherence remained a highly significant predictor of the long-term verbal recall score. Columns 6 and 7 accounted for all above-mentioned control variables in one model, and our main variable of interest, Δ*C*_*A*_, remained significantly correlated.

**Table 3:** Linear regression of long-term memory on Δ*C*.

	**Long-term verbal recall**						
	**1**	**2**	**3**	**4**	**5**	**6**	**7**
**A. ANTERIOR COHERENCE**							
Δ*C_*A*_*	−1.40^***^	−1.22^***^	−0.98^***^	−1.14^***^	−0.45^**^	−0.94^***^	−0.34^*^
	(0.31)	(0.25)	(0.28)	(0.24)	(0.20)	(0.23)	(0.18)
Minus trail-making Test A		0.08				−0.01	0.04
		(0.04)				(0.05)	(0.03)
Minus trail-making Test B			0.05^***^			0.04	0.01
			(0.02)			(0.03)	(0.02)
IST2000				0.10^***^		0.06^*^	−0.02
				(0.03)		(0.04)	(0.03)
Working memory (verbal, learning)					0.24^***^		0.23^***^
					(0.02)		(0.02)
Semi-partial *R*^2^ for Δ*C_*A*_*	0.28	0.24	0.18	0.23	0.08	0.17	0.06
Adjusted *R*^2^	0.28	0.33	0.41	0.39	0.74	0.41	0.76
**B. TOTAL COHERENCE**							
Δ*C_*T*_*	−1.13^***^	−0.94^***^	−0.85^***^	−0.89^***^	−0.46^**^	−0.81^***^	−0.39^**^
	(0.25)	0.26	(0.27)	(0.22)	(0.19)	0.23	0.17
Control variables (as above)	−	Yes	Yes	Yes	Yes	Yes	Yes
Semi-partial *R*^2^ for Δ*C_*T*_*	0.19	0.15	0.15	0.15	0.11	0.15	0.09
Adjusted *R*^2^	0.19	0.25	0.39	0.32	0.75	0.40	0.77
Number of individuals	40	40	40	40	40	40	40

*Robust standard errors in parentheses*.

* *p < 0.10*,

** *p < 0.05*,

*** *p < 0.01*.

*All specifications include a constant that was omitted from the table*.

*Each column represents one linear regression model with additional variables added. Control variables were added gradually, showing that the coefficient on the difference in coherence is robust enough to account for these factors. Columns 2–3 were adjusted for each of the three cognitive test measures of speed of processing and visual attention. Column 4 was adjusted for intelligence score (i.e., I-S-T-2000-R). Column 5 was adjusted for working memory using the 15-word pair learning score. Columns 6–7 was adjusted for all control variables in one model. The linear fits are depicted in Figure 2. The semi-partial R^2^ for the main explanatory variables are shown along the total adjusted R^2^*.

The same patterns of robust significant correlations were observed for total coherence of the brain and memory but explained a smaller proportion of the variability (semi-partial *R*^2^ = 0.19, *p* < 0.01). Table 3B shows the multiple linear regression models with a gradually increasing set of control variables when investigating Δ*C*_*T*_, with a raw parameter estimate of −1.13 (*p* < 0.01). Table 4 shows the results for Δ*C*_*A*_ and Δ*C*_*T*_ when controlling for visual attention as measured by RVP (columns 1, 3–4), birth weight and length. Controlling for sustained visual attention (RVP), our main explanatory variables remain significant. We also controlled for years of education (Table 4, columns 2–4). Education significantly correlated with long-term recall score, as expected, and had high explanatory power with a semi-partial *R*^2^ of 0.09 for the long-term recall data (*p* = 0.07). Nevertheless, Δ*C*_*A*_ remains a significant predictor of long-term recall, even controlling for education, and can explain an additional 14% of the variation in memory that neither of the control variables can explain (semi-partial *R*^2^ = 0.14, *p* < 0.01) for total coherence (Δ*C*_*T*_ semi-partial *R*^2^ = 0.09, *p* < 0.05). When controlling for the intelligence score, years of education lost significance, whereas the coefficients on the main explanatory variables (Δ*C*_*A*_ and Δ*C*_*T*_) dropped in absolute size but remained negative and significant.

**Table 4:** Linear regression of long-term memory and Δ*C*—alternative control variables.

	**Long-term verbal recall**			
	**1**	**2**	**3**	**4**
**A. ANTERIOR COHERENCE**				
Δ*C_*A*_*	−1.09^***^	−1.01^***^	−0.72^**^	−0.39^*^
	(0.37)	(0.37)	(0.33)	(0.19)
Minus trail-making Test A			−0.02	0.05
			(0.06)	(0.03)
Minus trail-making Test B			0.04	0.01
			(0.03)	(0.02)
RVP (A)	10.98		−2.45	−8.2
	(11.57)		(8.7)	(6.52)
IST2000			0.08^*^	0.00
			(0.04)	(0.03)
Working memory (verbal, learning)				0.23^***^
				(0.03)
Birth weight		−0.33	−0.03	0.13
		(1.39)	(1.47)	(0.71)
Birth length		0.07	−20.0	−5.49
		(31.98)	(32.12)	(15.68)
Years of education		0.06	0.06	−0.02
		(0.20)	(0.20)	(0.13)
Semi-partial *R*^2^ for Δ*C_*A*_*	0.15	0.14	0.09	0.07
Adjusted *R*^2^	0.13	0.15	0.21	0.71
**B. TOTAL COHERENCE**				
Δ*C_*T*_*	−0.95^***^	−0.73^**^	−0.63^*^	−0.34^*^
	(0.33)	(0.29)	(0.33)	(0.19)
Control variables (as above)		Yes	Yes	Yes
Semi-partial *R*^2^ for Δ*C_*T*_*	0.13	0.09	0.08	0.07
Adjusted *R*^2^	0.12	0.09	0.20	0.71
Number of individuals	35	35	35	35

*Robust standard errors in parentheses*.

* *p < 0.10*,

** *p < 0.05*,

*** *p < 0.01*.

*All specifications include a constant that was omitted from the table*.

*Linear regression of the long-term verbal recall and difference between monomodal and bimodal coherence (ΔC_A_) when controlling for bio-social factors, such as birth measures, years of education, and furthermore neurocognitive tests for speed of processing and attention. Each column represents one linear regression model. Control variables were added in a stepwise fashion, showing that the correlation between coherence and long-term memory scores was robust enough to account for these factors. Columns 1–3 were adjusted for sociodemographic factors, such as birth measures and years of education. Columns 2–3 control for each of the three cognitive test measures of speed of processing and visual attention, as well as the intelligence score (i.e., I-S-T-2000-R) and working memory using the 15-word pair learning score. The semi-partial R^2^ for the main explanatory variables are shown along the total adjusted R^2^*.

The Pearson correlations between the different memory scores and speed of processing, visual attention, frontal executive function, intelligence score, years of education, and birth measures are shown in Table 5. This table establishes that intelligence and memory is highly significantly correlated. Furthermore, we found that our measure of interest (Δ*C*_*A*_ and Δ*C*_*T*_) also are highly significantly correlated with memory but only borderline correlated (i.e., at the 10% significance level) with the intelligence score (not reported in the Table).

**Table 5:** Pearson correlations between different memory scores and speed of processing, visual attention, frontal executive function, intelligence score, years of education, and birth measures.

	**Long-term verbal recall**	**Working memory**			
	**Memory (recall)**	**Memory (learning)**	**PAL (total errors multiplied by −1, adjusted)**	**PRM (number correct)**	**SRM (number correct)**
Memory (learning)	0.86^***^	–			
PAL (total errors multiplied by −1, adjusted)	0.41^***^	0.34^**^	–		
PRM (number correct)	0.41^***^	0.45^***^	0.31^*^	–	
SRM (number correct)	0.23	0.20	0.09	0.26^*^	–
Minus trail-making Test A	0.40^***^	0.24^*^	0.26^*^	0.15^*^	0.33^**^
Minus trail-making Test B	0.56^***^	0.46^***^	0.39^**^	0.35^**^	0.34^**^
SDMT	0.31^*^	0.32^**^	0.38^**^	0.33^**^	0.18
RVP (number of total Hits)	0.18	0.29^*^	0.10	0.15	0.12
IST2000	0.49^***^	0.53^***^	0.27^*^	0.36^**^	0.16
Birth weight^a^	−0.26	−0.23	−0.04	0.26	−0.14
Birth length^a^	−0.25	−0.23	−0.02	0.16	−0.06
Years of education	0.39^**^	0.44^***^	0.23	0.18	−0.10
Number of individuals	40	40	40	40	40

*Robust standard errors in parentheses*.

* *p < 0.10*,

** *p < 0.05*,

*** *p < 0.01*.

*All specifications include a constant that was omitted from the table*.

a *Based on a sample of 36 subjects*.

*PAL, Paired Associates Learning; PRM, Pattern Recognition Memory; SRM, Spatial Recognition Memory; SOC, Stockings of Cambridge*.

Because education may be an outcome of intelligence (and *vice versa*), controlling for education in the regression models of intelligence and our EEG measures may generate a bias in the estimates when interpreted as causal effects of coherence on memory. This is further evidence in favor of the interpretation that Δ*C*_*A*_ contains information related to the biological cognitive mechanism that is not due to other factors, such as education.

Changing the time window from 100 to 1,000, and 6,000 ms in a total stimulation period of 6,000 ms, we still found significant negative correlation between memory and Δ*C*_*A*_, as well as Δ*C*_*T*_, though with a lower explanatory power (see Table 6).

**Table 6:** Linear regression of the long-term memory and difference between monomodal and bimodal coherence (Δ*C*) when investigating the effect of different time windows.

	**Long-term verbal recall**			
	**1,000 ms**		**6,000 ms**	
	**1**	**2**	**3**	**4**
**A. ANTERIOR COHERENCE**				
Δ*C_*A*_*	–0.41^*^	–0.53^***^	–0.22^*^	–0.28^***^
	(0.41)	(0.17)	(0.22)	(0.09)
Minus trail-making Test A		0.05^*^		−0.05^**^
		(0.03)		(0.03)
Minus trail-making Test B		0.01		0.00^*^
		(0.01)		(0.01)
RVP (A)		−6.21		−6.20
		(5.81)		(5.81)
IST2000		−0.00		−0.00
		(0.03)		(0.03)
Verbal learning score		0.24^***^		0.24^***^
		(0.03)		(0.03)
Birth weight		0.52		0.52
		(0.75)		(0.57)
Birth length		−12.64		−12.46
		(16.77)		(16.77)
Years of education		−0.04		−0.04
		(0.12)		(0.12)
Semi-partial *R*^2^ for Δ*C_*A*_*	0.02	0.16	0.02	0.16
Adjusted *R*^2^		0.74		0.74
	40	35	40	35
**B. TOTAL COHERENCE**				
Δ*C_*T*_*	–0.46^**^	–0.60^***^	–0.22^**^	–0.29^***^
	(0.41)	(0.18)	(0.20)	(0.09)
Semi-partial *R*^2^ for Δ*C_*T*_*	0.03	0.19	0.03	0.19
Adjusted *R*^2^		0.75		0.75
Number of individuals	40	35	40	35

*Robust standard errors in parentheses*.

* *p < 0.10*,

** *p < 0.05*,

*** *p < 0.01*.

*All specifications include a constant that was omitted from the table*.

*Linear regression of the long-term memory and difference between monomodal and bimodal coherence (ΔC) when investigating the effect of different time windows while controlling for sociodemographic factors, such as birth measures, years of education, and neurocognitive tests for speed of processing and attention as in Table 2. In Panel A we investigate the mono-to-bimodal coherence differences in the anterior part of the brain. In Panel B we investigate the mono-to-bimodal coherence differences in the total brain. Each column represents one linear regression model. Adding control variables, we find that the coefficient on coherence was robust enough to account for these factors. Columns 2 and 4 were adjusted for bio-social factors (i.e., birth measures and years of education), each of the three cognitive test measures of speed of processing and visual attention, as well as the intelligence score (i.e., I-S-T-2000-R) and working memory using the 15-word pair learning score. The semi-partial R^2^ for the main explanatory variables are shown along the total adjusted R^2^*.

**Figure 3:**
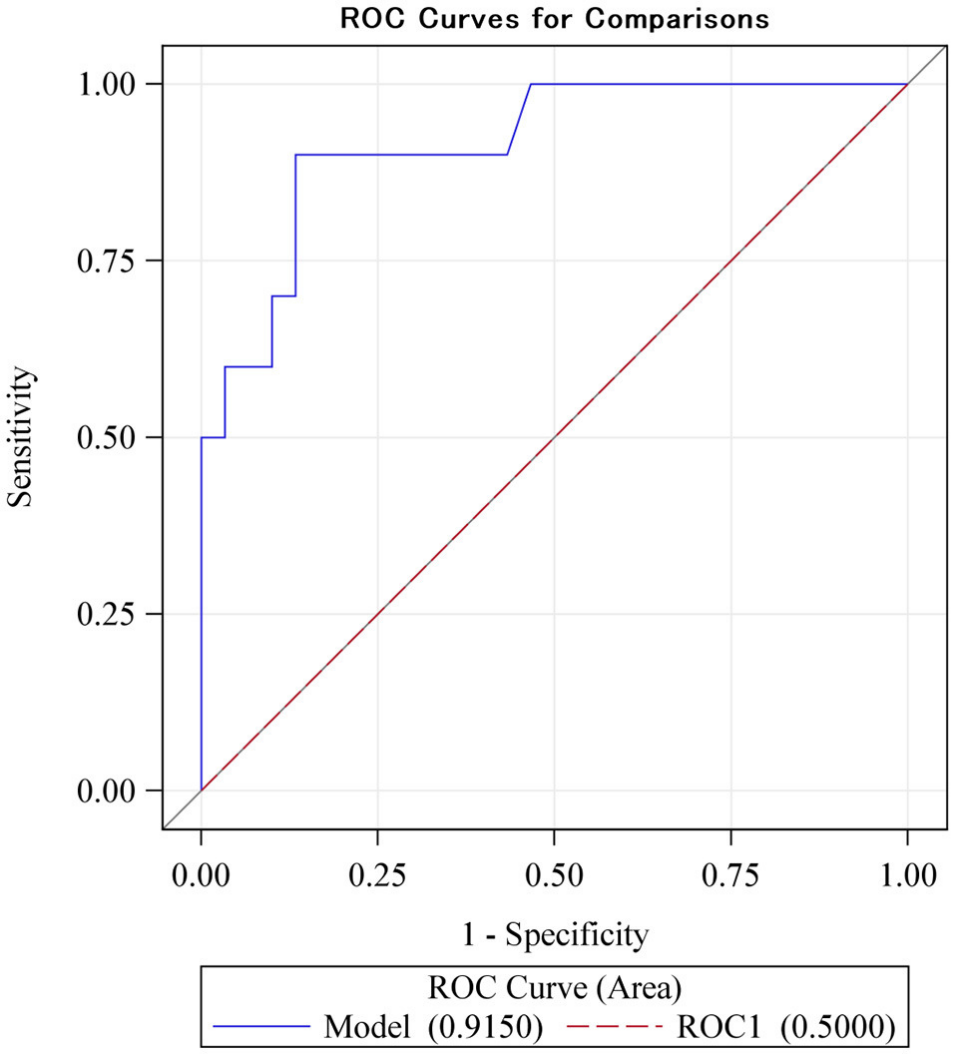
The ROC sensitivity curve of the main explanatory variable (difference between monomodal and bimodal gamma steady-state response) as a predictor of low intelligence scores (defined as lower than two-thirds of a standard deviation below the mean). With a cut-off value for Δ*C*_*A*_ of 0.25 the sensitivity for prediction of low memory is as high as 0.9 and the specificity is as high as 0.87 (Youden-index: 0.77). The ROC sensitivity is 92% (CI: 0.81–1.00).

Finally, we investigated the clinical prediction of low memory capacity, defined as two-thirds the SD below the mean for the population. With a cut-off value for Δ*C*_*A*_ of 0.25 the sensitivity for prediction of low memory is as high as 90% and the specificity is as high as 87% (Youden-index: 0.77). The raw ROC sensitivity of Δ*C*_*A*_ was 92% (CI: 0.81–1.00, Figure 3). This rather high sensitivity likely indicates that our EEG method has great promise as a diagnostic tool in cognitive function.

### Working memory and gamma coherence

#### Visual and verbal memory

Performing a similar analysis when investigating working memory, we found the same negative correlation between Δ*C*_*A*_ and Δ*C*_*T*_, though with lower explanatory power. Table 7 investigates four working memory tests and the difference in coherence for the total brain and the anterior part only. Table 7A, establishes that Δ*C*_*A*_ significantly correlated with verbal learning scores (*R*^2^ = 0.22, *p* < 0.01) and visual memory (i.e., PAL to assess episodic memory and learning rate, *R*^2^ = 0.08, *p* < 0.05, columns 3–4; PRM assessing visual recognition memory for patterns, *R*^2^ = 0.18, *p* < 0.01, columns 5–6; SRM assessing recognition memory for spatial location, *R*^2^ = 0.07, *p* = 0.07, columns 7–8). The coefficients remained significantly negative even when controlling for speed of processing (Trail-making Test A), executive function (Trail-making Test B), and intelligence score. On the other hand, Δ*C*_*T*_ lost significance with respect to verbal memory when using the 15-word paired associates learning while controlling for the four main control variables (column 2).

**Table 7:** Linear regression of different working memory test scores on Δ*C*—controlling for neurocognitive test scores for speed of processing and attention.

	**VERBAL LEARNING AND CANTAB MEASURES**										
	**Verbal memory**		**Visual memory**						**Executive function (planning)**		
	**Memory (learning)**		**PAL (total errors multiplied by** −**1, adjusted)**		**PRM (number correct)**		**SRM (number correct)**		**SOC (problems solved in min. move)**		
	**1**	**2**	**3**	**4**	**5**	**6**	**7**	**8**	**9**	**10**	**11**
**A. ANTERIOR COHERENCE**											
Δ*C_*A*_*	−4.04^***^	−2.63^***^	−6.66^**^	−3.44^**^	−0.75^***^	−0.54^**^	−0.38^*^	−0.42^**^	−0.45	−0.51^*^	−0.60^**^
	(1.13)	(0.97)	(3.28)	(3.32)	(0.21)	(0.24)	(0.21)	(0.21)	(0.28)	(0.29)	(0.28)
Minus TA		−0.25		−0.19		−0.05		0.03			−0.07^**^
		(0.19)		(0.58)		(0.06)		(0.04)			(0.03)
Minus TB		0.13		0.40		0.02		0.01			
		(0.10)		(0.31)		(0.02)		(0.02)			
I-S-T 2000-R		0.33^***^		0.156		0.04		−0.01			0.03
		(0.11)		(0.44)		(0.03)		(0.03)			(0.02)
SOC (thinking time, 5 moves)										−0.00^***^	−0.00^***^
										(0.00)	(0.00)
Semi-partial *R*^2^ for Δ*C_*A*_*	0.22	0.12	0.08	0.02	0.18	0.10	0.07	0.03	0.08	0.12	0.15
Adjusted *R*^2^	0.22	0.37	0.08	0.9	0.15	0.20	0.14	0.06		0.21	0.26
**B. TOTAL COHERENCE**											
Δ*C_*T*_*	−2.74^**^	−1.83^*^	−6.05^*^	−4.28	−0.57^**^	−0.46^*^	−0.53^**^	−0.45^**^	−0.35	−0.34	−0.38
	(1.05)	(1.00)	(3.01)	(3.47)	(0.28)	(0.27)	(0.24)	(0.22)	(0.23)	(0.24)	(0.25)
Control variables (as above)	−	Yes	−	Yes	−	Yes	−	Yes			
Semi-partial *R*^2^ for Δ*C_*T*_*	0.10	0.07	0.07	0.04	0.10	0.08	0.13	0.10	0.05	0.05	0.07
Adjusted *R*^2^		0.33		0.10		0.18		0.13		0.16	0.18
Number of Individuals	40	40	40	40	40	40	40	40	39	39	39

*Robust standard errors in parentheses*.

* *p < 0.10*,

** *p < 0.05*,

*** *p < 0.01*.

*All specifications include a constant that was omitted from the table*.

*Linear regression of the different working memory tests on the difference between monomodal and bimodal coherence (ΔC), controlling for neurocognitive tests for speed of processing and attention as in Table 2. In Panel A we investigate the mono-to-bimodal coherence differences in the anterior part of the brain (ΔC_A_). In Panel B we investigate the mono-to-bimodal coherence differences in the total brain (ΔC_T_). Each column represents one linear regression model. When control variables were added, we find that the coefficient on the difference in coherence was robust enough to account for these factors. Columns 2, 4, 6, 8, 10, and 11 were adjusted for each of the three cognitive test measures of speed of processing and executive function, as well as the intelligence score (i.e., I-S-T-2000-R). The semi-partial R^2^ for the main explanatory variables are shown along the total adjusted R^2^. PRM, Pattern Recognition Memory; SRM, Spatial Recognition Memory; PAL, Paired Associates Learning; SOC, Stockings of Cambridge*.

#### Executive function

In columns 9–11 of Table 7 we investigated the correlation between executive function and Δ*C*_*A*_ and Δ*C*_*T*_. The SOC measure used is the number of problems solved in the minimum possible amount of moves and tested planning and executive function. This measure is negatively associated with the difference in coherence (Δ*C*_*A*_: *R*^2^ = 0.08, *p* = 0.11; Δ*C*_*T*_: *R*^2^ = 0.05, *p* = 0.15). When further controlling for SOC thinking time in five moves, Δ*C*_*A*_ were significant at the 10% level (Δ*C*_*A*_: *p* = 0.09). Finally, when adding all four main control variables, Δ*C*_*A*_ and Δ*C*_*T*_ were significant for 5% (Δ*C*_*T*_: *p* = 0.14, Δ*C*_*A*_: *p* < 0.05).

We also controlled for sleep habits and fatigue with potentially confounding effects on presently measured memory scores (see Table 8). The coefficients of interest remained significant in all specifications, further solidifying our conclusion that the association between EEG measurements and memory represents a constituent relationship. In particular, because memory scores can be affected by a range of mental factors, such as fatigue and sleep habits, we controlled for the measurement of these factors. Including the MFI and PSQI scores as control variables in the analysis, we found that the difference between monomodal and bimodal stimulation (Δ*C*_*A*_ and Δ*C*_*T*_) remained significantly correlated with long-term memory (Table 8).

**Table 8:** Linear regression of long-term memory on Δ*C*—when controlling for sleep habits and fatigue.

	**Long-term recall (verbal)**					
	**1**	**2**	**3**	**4**	**5**	
**A. ANTERIOR COHERENCE**						
Δ*C_*A*_*	−1.41^***^	−1.48^***^	−1.26^***^	−1.52^***^	−1.42^***^	−1.49^***^
	(0.36)	(0.39)	(0.33)	(0.37)	(0.34)	(0.44)
Mental fatigue		0.22			0.48^**^	0.77^*^
		(0.49)			(0.33)	(0.40)
Reduced motivation		0.02			0.043	−0.05
		(0.51)			(0.7)	(0.40)
Reduced activity		−0.48^*^			−0.62^***^	−0.68^***^
		(0.25)			(0.18)	(0.23)
Physical fatigue score		0.22			0.37	0.34
		(0.41)			(0.41)	(0.38)
General fatigue score		−0.06			−0.24	−0.37
		(0.31)			(0.47)	(0.27)
Epworth sleepiness scale			0.24		0.28^**^	0.30^**^
			(0.15)		(0.13)	(0.13)
Hours of sleep				0.13	0.42	0.27
				(0.56)	(0.56)	(0.58)
Pittsburgh sleep quality index				0.26	0.51^**^	0.42^**^
				(0.29)	(0.21)	(0.20)
Semi-partial *R*^2^ for Δ*C_*A*_*	0.28	0.29	0.24	0.30	0.29	0.27
**B. TOTAL COHERENCE**						
Δ*C_*T*_*	−1.27^***^	−1.22^***^	−1.14^***^	−1.42^***^	−1.27^***^	−1.41^***^
	(0.31)	(0.30)	(0.72)	(0.28)	(0.30)	(0.45)
Semi-partial *R*^2^ for Δ*C_*T*_*	0.20	0.21	0.18	0.24	0.30	0.25
Number of individuals	35	35	35	35	35	35

*Robust standard errors in parentheses*.

* *p < 0.10*,

** *p < 0.05*,

*** *p < 0.01*.

*All specifications include a constant that was omitted from the table*.

*Linear regression of the long-term memory and difference between monomodal and bimodal coherence (ΔC) when controlling for sleep habits and Multidimensional Fatigue Inventory. In Panel A we investigate the mono-to-bimodal coherence differences in the anterior part of the brain. In Panel B we investigate the mono-to-bimodal coherence differences in the total brain. Each column represents one linear regression model. Control variables were added in a stepwise fashion, showing that the regression between coherence and long-term verbal recall scores was robust enough to account for these factors. Columns 2 and 4 were adjusted for sociodemographic factors (i.e., birth measures and years of education), each of the three cognitive test measures for speed of processing and visual attention, as well as the intelligence score (i.e., I-S-T-2000-R) and working memory using the 15-word pair learning score. The semi-partial R^2^ for the main explanatory variables are shown along the total adjusted R^2^*.

Similarly, when using working memory measures as the outcomes, we controlled for sleep habits, fatigue, birth measures, and years of education, finding again that our main explanatory variable remained significantly and robustly associated with these memory measures (Table 9).

**Table 9:** Linear regression of working memory on Δ*C*—controlling for sleep habits, fatigue, birth measures, and years of education.

	**WORKING MEMORY**									
	**Verbal memory Memory (learning)**		**PAL (total errors multiplied by** − **1, adjusted)**		**Visual memory PRM (number correct)**		**SRM (number correct)**		**Executive function SOC (problems solved in min. move)**	
	**1**	**2**	**3**	**4**	**5**	**6**	**7**	**8**	**9**	**10**
**A. ANTERIOR COHERENCE**										
Δ*C_*A*_*	−2.64^*^	−2.43	−7.88^**^	−9.13^**^	−0.90^***^	−0.90^***^	−0.70^***^	−0.80^***^	−0.74^**^	−0.93^**^
	(1.35)	(1.50)	(3.53)	(4.19)	(0.27)	(0.34)	(0.24)	(0.22)	(0.30)	(0.37)
Birth measures and years of education	Yes	No	Yes	No	Yes	No	Yes	No	Yes	No
Fatigue, Epworth sleepiness scale, and PSQI	No	Yes	No	Yes	No	Yes	No	Yes	No	Yes
Semi-partial *R*^2^ for Δ*C_*A*_*	0.09	0.08	0.08	0.15	0.20	0.22	0.16	0.22	0.14	0.18
**B. TOTAL COHERENCE**										
Δ*C_*T*_*	−1.13	−2.72^**^	−6.27	−8.94^**^	−0.55^*^	−0.82^***^	−0.88^***^	−0.84^***^	−0.58^**^	−0.73^**^
	(1.20)	(1.24)	(3.99)	(4.28)	(0.28)	(0.28)	(0.26)	(0.22)	(0.25)	(0.33)
Control variables (as above)										
Semi-partial *R*^2^ for Δ*C_*T*_*	0.02	0.10	0.05	0.15	0.08	0.19	0.27	0.25	0.09	0.12
Number of Individuals	35	35	35	35	35	35	35	35	35	35

*Robust standard errors in parentheses*.

* *p < 0.10*,

** *p < 0.05*,

*** *p < 0.01*.

*All specifications include a constant that was omitted from the table*.

*PAL, Paired Associates Learning; PRM, Pattern Recognition Memory; SRM, Spatial Recognition Memory; SOC, Stockings of Cambridge*.

#### Left, right, intra-, and inter-hemispheric differences

Subdividing the differences in total coherence into right and left hemispheric coherence, we found that the coherence at the language-specific left hemisphere significantly negatively correlated with long-term memory with higher explanatory power than the right side (parameter estimate −1.69, *SD* 0.49, *p* < 0.01, *R*^2^ = 0.22, results available upon request), and significant on 10% level in the anterior part of the brain (parameter estimate −1.11, *SD* 0.57, *p* = 0.06, *R*^2^ = 0.09).

When investigating the intra- and inter-hemispheric coherences in the anterior part of the brain, we found a joint negative interaction effect of the intra- and inter-hemispheric coherence but it was not significant, suggesting that intra- and inter-hemispheric coherence jointly determine memory.

#### Additional robustness checks

All of the findings were robust to including an additional four observations with missing information on birth measures.

Furthermore, the results were robust to controlling for the difference in power at 36 Hz in the frontal and pre-frontal area, defined analogously to the difference in coherence. In addition, the difference in power was not correlated with the difference in coherence.

Finally, in our sample, 36 subjects where right handed while four subjects were left handed. Controlling for handedness by including a dummy capturing this in the raw main regression (corresponding to Table 3, column 1) we found the same qualitative conclusion (for Δ*C*_*A*_: coefficient = −1.39, *p* < 0.01, semi-partial *R*^2^ = 0.28; for Δ*C*_*T*_: coefficient = −1.11, *p* < 0.01, *R*^2^ = 0.19). Furthermore, when also controlling for the intelligence and trail making scores (corresponding to Table 3, column 6) we again reached the same qualitative conclusion (for Δ*C*_*A*_: coefficient = −0.92, *p* < 0.01, semi-partial *R*^2^ = 0.17; Δ*C*_*T*_: coefficient = −0.80, *p* < 0.01, *R*^2^ = 0.14). Moreover, restricting the sample to right-handed subjects, we also find the same qualitative conclusion with an increased semi-partial *R*^2^ (for Δ*C*_*A*_: coefficient = −1.44, *p* < 0.01, semi-partial *R*^2^ = 0.35; for Δ*C*_*T*_: coefficient = −1.32, *p* < 0.01, semi-partial *R*^2^ = 0.29) and this changed only a little when controlling for intelligence and trail making scores (for Δ*C*_*A*_: coefficient = −1.00, *p* < 0.01, semi-partial *R*^2^ = 0.27; Δ*C*_*T*_: coefficient = −0.97, *p* < 0.01, *R*^2^ = 0.27).

#### Analysis with auditory responses

We also investigated differences in coherence between monomodal and bimodal stimulation in the anterior part as well as in the total brain for the *auditory* response (Table 10). The auditive measure is defined analogously to the visual measure, and denoted Δ*C'*_*A*_. Importantly, while the raw correlation between this new auditory measure and memory is negative, similarly to the visual measure, we find that only the visual measure is statistically significant at the 5% significance level. The auditory measure for the whole brain is statistically significant only at the 10% significance level. In addition, we find that the correlation between the visual coherence measure and memory performance remains significantly correlated even when including the auditory measure as a control variable in the model.

**Table 10:** Analysis with auditory explanatory variable.

	**Long-term verbal recall**			
	**1**	**2**	**3**	**4**
**A. ANTERIOR COHERENCE**				
Δ*C_*A*_*			−1.39^***^	−0.95^***^
			(0.31)	(0.22)
Δ*C'_*A*_*	−0.31	0.35	−0.06	0.39
	(0.47)	(0.43)	(0.46)	(0.40)
Main control variables		Yes		Yes
Semi-partial *R*^2^ for Δ*C_*A*_*			0.28	0.18
Semi-partial *R*^2^ for Δ*C'_*A*_*	0.01	0.02	0.00	0.03
**B. TOTAL COHERENCE**				
Δ*C_*T*_*			−0.95^***^	−0.70^**^
			(0.33)	(0.35)
Δ*C'_*T*_*	−0.94^*^	−0.64	−0.37	−0.24
	(0.48)	(0.41)	(0.55)	(0.53)
Main control variables		Yes		Yes
Semi-partial *R*^2^ for Δ*C_*T*_*			0.11	0.09
Semi-partial *R*^2^ for Δ*C'_*T*_*	0.11	0.07	0.01	0.01

*Robust standard errors in parentheses*.

* *p < 0.10*,

** *p < 0.05*,

*** *p < 0.01*.

*All specifications include a constant that was omitted from the table. Main Control Variables: Minus Trail-making Test A, Minus Trail-making Test B, IST2000. In both panels and all columns, the number of observations (individuals) is 40*.

*Linear regression of the long-term verbal recall and difference between monomodal and bimodal coherence for the visual measure (ΔC_A_) and the auditive measure (ΔC'_A_)*.

In particular, the results of the auditive analysis for the anterior brain region can be found in Table 10A. In all four columns, the auditive measure is insignificant. Interestingly, when including the visual measure, it remains highly statistically significant when not including any control variables (column 3) and when including all the baseline control variables (column 4). Furthermore, the results of the auditive analysis for the whole brain region can be found in Table 10B. Column 1 of that panel indicates that the auditive measure is negatively correlated with long-term verbal recall (significant only at the 10% significance level). Column 2 shows that the estimate diminishes numerically and is no longer significant at even the 10% level when we include the main control variables. Columns 4 and 5 establishes that the visual measure remains significantly negatively correlated with long-term verbal recall, even when including the auditive measure and the control variables in the model.

## Discussion

### General discussion

This present study finds that a *passive* EEG stimulation response can explain memory capacity. In contrast, previous studies have typically investigated memory performance in relation to the EEG stimulation response obtained during an *active* cognitive task. Furthermore, previous studies have typically been based on transient EEG gamma activity and p300 analysis (Tallon-Baudry et al., 1998; Fell et al., 2001; Gruber et al., 2002; Ricciardi et al., 2006; Missonnier et al., 2007), while this present study has focused on steady-state evoked potentials. To the best of our knowledge, this is the first study of the correlation between individual differences in coherence from bimodal to monomodal stimulation and long-term memory, as well as working memory, using non-task steady-state sensory stimulation.

It is interesting to note that the correlation between our measures of interest and memory is robust to controlling for intelligence, indicating that the correlations are not entirely caused by variations in intelligence across subjects. These findings are consistent with a notion that memory is more related to brain synchronization than intelligence. Meanwhile, it is also worth noting that the coefficient on the measures of interest does become smaller in absolute magnitude when intelligence is controlled for, suggesting that at least some of the correlation can be attributed to intelligence effects. Future studies may be able to shed light on the interaction between memory, intelligence, and the EEG measures presented here.

We focused on the coherence of the total brain, with an emphasis on the frontal lobe, because the frontal and prefrontal region of the brain have been shown to have the most pronounced associations with cognitive processes. These result were found using both functional magnetic resonance imaging (Diwadkar et al., 2000; Curtis, 2006; Klingberg, 2006; Ricciardi et al., 2006), electroencephalography (Keil et al., 1999; McEvoy et al., 2001; Sauseng et al., 2004), transcranial magnetic stimulation (Oliveri et al., 2001; Rossi et al., 2004), and lesion studies (Carlesimo et al., 2001). Furthermore, Alzheimer's disease patients exhibit reduced fronto-(temporo-)parietal EEG coherence in mainly the theta, alpha, and gamma frequencies (Stam et al., 2002; Babiloni et al., 2004, 2016; Başar et al., 2016).

Our findings on the hemispheric sub-division is consistent with earlier studies, which found increased activity over the left hemisphere for the language condition and over the right hemisphere for the non-language condition (Eulitz et al., 1996; Babiloni et al., 2004; Ihara and Kakigi, 2006; Davis et al., 2008). A stronger correlation between left hemisphere coherences and verbal memory, compared to the correlation for the right hemisphere, was indicated in our results. This finding is also consistent with research showing that words evoke more cortical gamma oscillations than pseudo-words (Pulvermüller et al., 1995). Interestingly, patients with Alzheimer's disease have been shown to have impaired interhemispheric function (Babiloni et al., 2004). We found a joint negative but insignificant interaction effect of the intra- and inter-hemispheric coherence, suggesting that intra- and inter-hemispheric coherence jointly determine memory.

We focus on gamma activity since it has been associated with memory performance in the existing literature. For example, some studies using working-memory tests find that when subjects actively memorize visual stimuli, it induces more gamma activity than otherwise (Tallon-Baudry et al., 1998). Other studies have also positively correlated gamma activity with learning and memory (Miltner et al., 1999; Fell et al., 2001; Gruber et al., 2002). More broadly, gamma frequency activity has been found to correlate with multiple cognitive phenomena that may be related to memory. For example, gamma band activity has been shown to reflect binding processes (Gray et al., 1989; Müller et al., 1996; Tallon-Baudry and Bertrand, 1999), facial recognition (Keil et al., 1999), ambiguous visual stimuli (Tallon-Baudry et al., 1996, 1997, 1998; Rodriguez et al., 1999), detection of meaningfulness in autostereoscopic pictures consisting of random dot patterns (Revonsuo et al., 1997), as well as auditive stimuli (Pantev et al., 1991).

Furthermore, it has been found that attention to stimuli enhance the gamma response (Tiitinen et al., 1993; Eulitz et al., 1996; Herrmann et al., 2004b). Cognitive processes, such as the speed of manual reaction, alertness, and stress, also modulate gamma activity (Haig et al., 1999). However, the fact that the present results are robust to controlling for measures of attention, fatigue, speed of processing, among other controls, indicate that these potential factors are not driving the presents results.

Since, multiple oscillatory responses are associated with integrative brain functions, and oscillations in other frequency band are therefore likely as important as the oscillations in the gamma band, it is entirely possible that associations between mono-to-bimodal differences in coherence and memory can be found for other frequency bands (e.g., Klimesch, 1999; Başar et al., 2001; Hanslmayr et al., 2008, 2011, 2014; Staudigl and Hanslmayr, 2013; Hanslmayr and Staudigl, 2014; Roux and Uhlhaas, 2014). Furthermore, in light of the fact that opposite correlations between memory performance and brain activity has been found for the alpha and beta bands vs. theta and gamma bands (Hanslmayr et al., 2008), we could hypothesize that opposite associations between mono-to-bimodal differences and memory exists for the alpha and beta bands. Further studies are needed to investigate alternative frequency bands.

Furthermore, a larger spectrum of frequency bands included in analyses could potentially increase the precision with which memory capacity can be predicted on the basis of EEG recordings. We find that Δ*C*_*A*_ have a high sensitivity and specificity for prediction of low memory capacity, but it is possible that incorporating information based on additional frequencies will add more information that can help generate even better predictions.

Moreover, in addition to studying additional frequencies, it would be interesting to investigate more deeply the functional form of the relationship, and in particular if the relationship is truly linear.

In addition, we focus especially on the anterior part of the brain, since it has been shown that theta and gamma oscillations occur separately at frontal and prefrontal areas, simultaneously with strong phase-coupling during short-term memory processing (Jacobs et al., 2006). An pronounced anterior effect may reflect a functional link between the prefrontal areas and the gyrus cinguli, which are important for memory functions (Schack et al., 2002). In light of the importance of the fronto-parietal neural circuitry for cognitive function, it is interesting to note that the anterior-posterior coherence measure is also a significant predictor of memory performance, although its explanatory power as measured by its partial *R*^2^ is lower than that of the anterior measure.

We used a second incoming auditory stimulus in a combined passive audio-visual stimulation setup to investigate whether different evoked gamma responses interfere with each other and result in better description of the brain's synchronicity capacity, thereby gaining a better picture of the neural network connectivity of the thalamocortical network. While the use of passive stimulation is a central part of this research, it would be interesting to investigate if our explanatory variable has additional explanatory power when measured in a task-based setting. Future research could therefore attempt to perform a similar analysis using data recorded in a task-based setting. Likewise, future research could investigate if the same associations can be found using other stimulation frequencies.

In light of the correlations between cognitive processes and gamma activity, it is reassuring that our present results held up to controlling for measures of mental fatigue, sleep habits, and speed of processing, as well as visual attention. Overall, our findings suggest that brain gamma synchronization is associated with memory.

Our statistical analysis is designed to use the intra-individual differences between measures of monomodal and bimodal stimulation whereby we account for potential confounding factors that would affect both measures proportionally, such as head morphology or technical measurement error (e.g., the fact that the impedance may differ between electrodes).

## Limitations

While this study is the first to investigate a correlation between memory and individual differences in coherence using non-task steady state sensory stimulation and controlling for a number of possible confounders, it does suffer from a number of drawbacks.

First, it is impossible to determine in the present setup whether subjective factors such as arousal, individual variation in brain activity over the daily cycle, or individual day-specific factors such as stress or nervousness may affect the findings. However, the study is designed to mitigate these problems as much as possible in a single-measurement-session setup. In particular, the fact that the measure of interest is defined as an individual difference, implies that omitted factors that have a constant effect on the level of the brain responses in the monomodal and bimodal stimulations are automatically accounted for. For example, if the level of arousal of the individual at the time of measurement has a constant effect on each of the coherence measures that goes into the calculation of the differenced measure, this effect will not play a role in the regression analyses. Furthermore, we control statistically for as many observed factors as possible. Notably, we control for self-reported sleep habits and fatigue using control variables for mental fatigue, motivation, activity level, physical fatigue, general fatigue, sleepiness, hours of sleep, and sleep quality (Table 8).

Second, the fact that the participants were tested throughout the day, may have led to tiredness among some participants. It is not known to what extend this could have affected our results. For example, it is possible that our findings are specific to these conditions. Future studies could alleviate this concern by randomizing the order and timing of measurements. This would allow the researcher to control for the time-of-day of the EEG-measurement. Furthermore, it could be useful to identify the within-subject variability in the measure of interest by obtaining it multiple times a day and over several days.

Third, we cannot generalize our results to females or different age groups. Further studies are needed to investigate if the results presented here can also be found in other demographic groups. It will also be interesting to investigate a greater sample size with a wider span of levels of cognitive function. Furthermore, while we focused on gamma activity due to its association with both higher cognitive function and synchronicity, the same associations as those reported here could very well exist for other frequency bands, and future studies could therefore try to replicate our findings for other stimulation frequencies.

Finally, while we focused on gamma activity due to its association with both higher cognitive function and synchronicity, the same associations as those reported here could very well exist for other frequency bands, and future studies could therefore try to replicate our findings for other stimulation frequencies.

Furthermore, it would be interesting to investigate the robustness of our results with respect to alternative setups. For example, we referenced the signals to the mastoid electrodes out of concern for clinical relevance, but future research could investigate if this is a necessary condition for our findings, or if the results can also be established with alternative techniques, such as common average referencing or REST (Yao, 2001; Qin et al., 2010).

While the present method could predict current low memory performance with a high sensitivity, it was not yet possible to study later changes in cognitive function. Since the Metropolit cohort has been measured cognitively throughout life and will be continued to be measured in the coming years, we will be able to study the usability of the present method to predict changes in cognitive function.

## Conclusion

We found evidence that the difference in a subject's total brain coherence from the monomodal to the bimodal stimulation significantly correlated with long-term memory performance. This correlation was pronounced for the anterior region of the brain. In addition, the correlation between Δ*C* and long-term memory remained when controlling for working memory and a wide range of potentially confounding factors, including intelligence, length of education, and speed of processing. Furthermore, Δ*C*_*A*_ had a sensitivity of 90% and a specificity of 87% for the detection of low memory capacity (two-thirds of the SD below the mean). Finally, we found that Δ*C*_*A*_ is also associated with other types of memory, namely episodic memory, visual recognition memory, and spatial memory, and that these additional correlations are robust enough to control for a range of confounding factors.

## Author contributions

The author substantial contributions to the conception or design of the work (AH); or the acquisition (AH), analysis (AH), or interpretation of data for the work (AH, EM, MO, BF, ML, and KB); and Drafting the work (AH) or revising it critically for important intellectual content (AH, EM, MO, BF, ML, and KB); and Final approval of the version to be published (AH, EM, MO, BF, ML, and KB); and Agreement to be accountable for all aspects of the work in ensuring that questions related to the accuracy or integrity of any part of the work are appropriately investigated and resolved (AH, EM, MO, BF, ML, and KB).

### Conflict of interest statement

The authors declare that the research was conducted in the absence of any commercial or financial relationships that could be construed as a potential conflict of interest.

## Footnotes

^1^Note that this methodology also accounts for potential measurement issues that are affecting the coherence of either stimulation in an additive fashion and of the same magnitude. For example, this methodology may correct for issues measurement related to volume conduction or reference electrode effects.

## Abbreviations

ACE: Addenbrooke's cognitive examination
CANTAB: Cambridge Neuropsychological Test Automated Battery
CI: Confidence interval
EEG: Electroencephalography
ECG: Electrocardiogram
EMG: electromyography
EOG: Electrooculography
I-S-T 2000-R: Intelligenz-Struktur-Test 2000-R
Metropolit: Metropolit Danish Male Birth Cohort
MMSE: Mini-mental state examination
MOT: Motor screening task
ΔCA, Monomodal to bimodal stimulation coherence difference: anterior
ΔCT, Monomodal to bimodal stimulation coherence difference: total
SDMT: Symbol-digit modalities test
RTI: Reaction time
RVP: Rapid visual processing
SOC: Stockings of Cambridge
PAL: Paired associates learning
PRM: Pattern recognition memory
SRM: Spatial recognition memory.
